# Crystal structure of 4-[(2,4-di­chloro­phen­yl)(5-hy­droxy-3-methyl-1-phenyl-1*H*-pyrazol-4-yl)meth­yl]-5-methyl-2-phenyl-2,3-di­hydro-1*H*-pyrazol-3-one

**DOI:** 10.1107/S2056989015017880

**Published:** 2015-09-30

**Authors:** Balbir Kumar, Hitesh Mahajan, Satya Paul, Rajni Kant, Vivek K. Gupta

**Affiliations:** a Post-Graduate Department of Physics & Electronics, University of Jammu, Jammu Tawi 180 006 , India; b Department of Chemistry, University of Jammu, Jammu Tawi 180 006 , India

**Keywords:** crystal structure, pyrazolone, hydrogen bonding, π–π inter­actions

## Abstract

In the title compound C_27_H_22_Cl_2_N_4_O_2_, the pyrazol-5-ol ring makes a dihedral angle of 34.80 (11)° with the phenyl ring to which it is bound, while the pyrazolone ring is inclined at 34.34 (12)° to its attached phenyl ring. In the crystal, N—H⋯O and C—H⋯Cl hydrogen bonds link the mol­ecules into chains along [010]. Inter­molecular π–π inter­actions are observed between the pyrazolone ring and the phenyl ring bound to the pyrazol-5-ol ring system [centroid–centroid separation = 3.916 (2) Å].

## Related literature   

For the biological activity of bis-pyrazolo­nes, see: Park *et al.* (2005 ▸), and for their applications see: Bailey *et al.* (1985 ▸); Rosiere & Grossman (1951 ▸); Mahajan *et al.* (1991 ▸); Chauhan *et al.* (1993 ▸); Hamama *et al.* (2001 ▸). For the synthesis of similar compounds, see: Bhardwaj *et al.* (2015 ▸); Niknam & Mirzaee (2011 ▸). For related structures, see: Sharma *et al.* (2014 ▸).
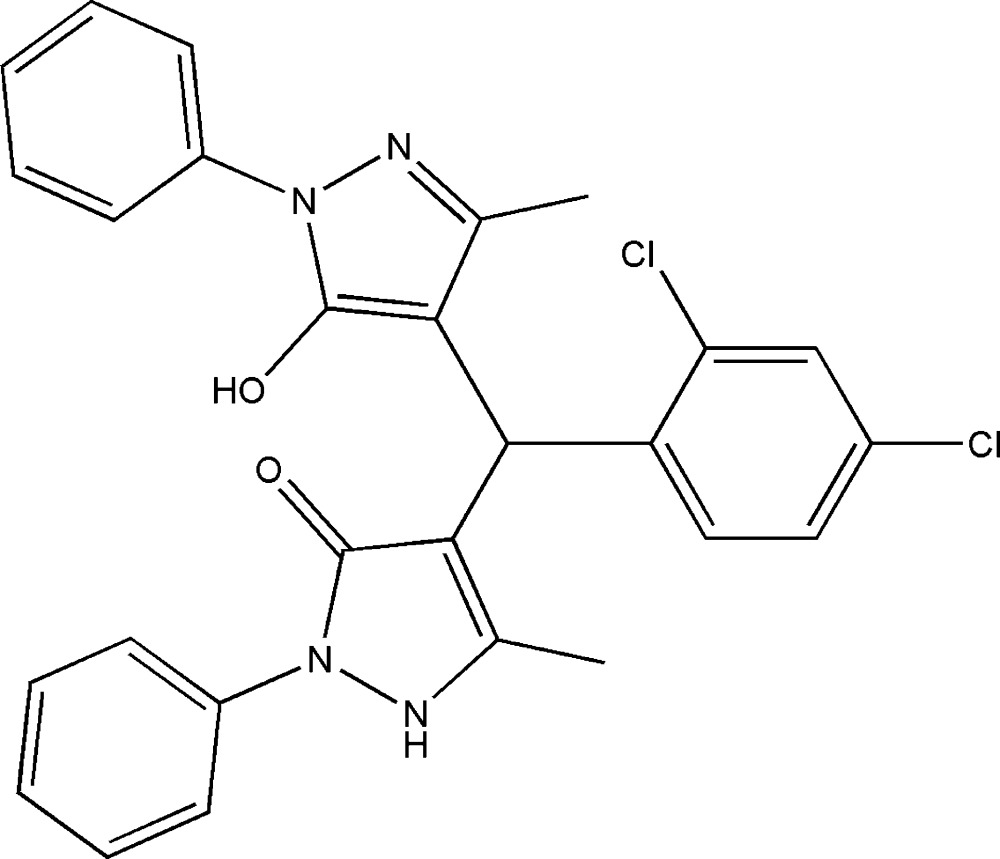


## Experimental   

### Crystal data  

C_27_H_22_Cl_2_N_4_O_2_
*M* *_r_* = 505.39
Monoclinic 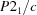

*a* = 19.8321 (19) Å
*b* = 7.8574 (5) Å
*c* = 16.3416 (16) Å
β = 106.815 (10)°
*V* = 2437.6 (4) Å^3^
*Z* = 4
Mo *K*α radiation
μ = 0.30 mm^−1^
*T* = 293 K
0.30 × 0.20 × 0.20 mm

### Data collection  

Agilent Xcalibur, Sapphire3 diffractometer
Absorption correction: multi-scan (*CrysAlis PRO*; Agilent, 2013 ▸) *T*_min_ = 0.751, *T*_max_ = 1.000
9479 measured reflections
4775 independent reflections
2524 reflections with *I* > 2σ(*I*)
*R*_int_ = 0.042

### Refinement  

*R*[*F* ^2^ > 2σ(*F* ^2^)] = 0.060
*wR*(*F* ^2^) = 0.174
*S* = 1.00
4775 reflections
318 parameters
H-atom parameters constrained
Δρ_max_ = 0.29 e Å^−3^
Δρ_min_ = −0.30 e Å^−3^

### 

Data collection: *CrysAlis PRO* (Agilent, 2013 ▸); cell refinement: *CrysAlis PRO*; data reduction: *CrysAlis PRO*; program(s) used to solve structure: *SHELXS97* (Sheldrick, 2008 ▸); program(s) used to refine structure: *SHELXL97* (Sheldrick, 2008 ▸); molecular graphics: *ORTEP-3 for Windows* (Farrugia, 2012 ▸); software used to prepare material for publication: *PLATON* (Spek, 2009 ▸).

## Supplementary Material

Crystal structure: contains datablock(s) I, New_Global_Publ_Block. DOI: 10.1107/S2056989015017880/sj5478sup1.cif

Structure factors: contains datablock(s) I. DOI: 10.1107/S2056989015017880/sj5478Isup2.hkl

Supporting information file. DOI: 10.1107/S2056989015017880/sj5478Isup3.cml

**Fig. 1.**
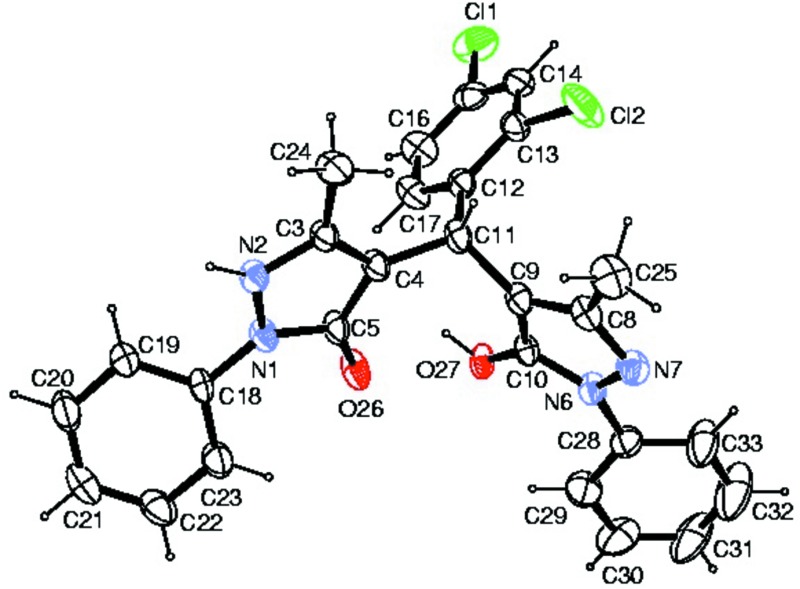
ORTEP view of the molecule with the atom-labeling scheme. Displacement ellipsoids are drawn at the 40% probability level. H atoms are shown as small spheres of arbitrary radii.

**Fig. 2.**
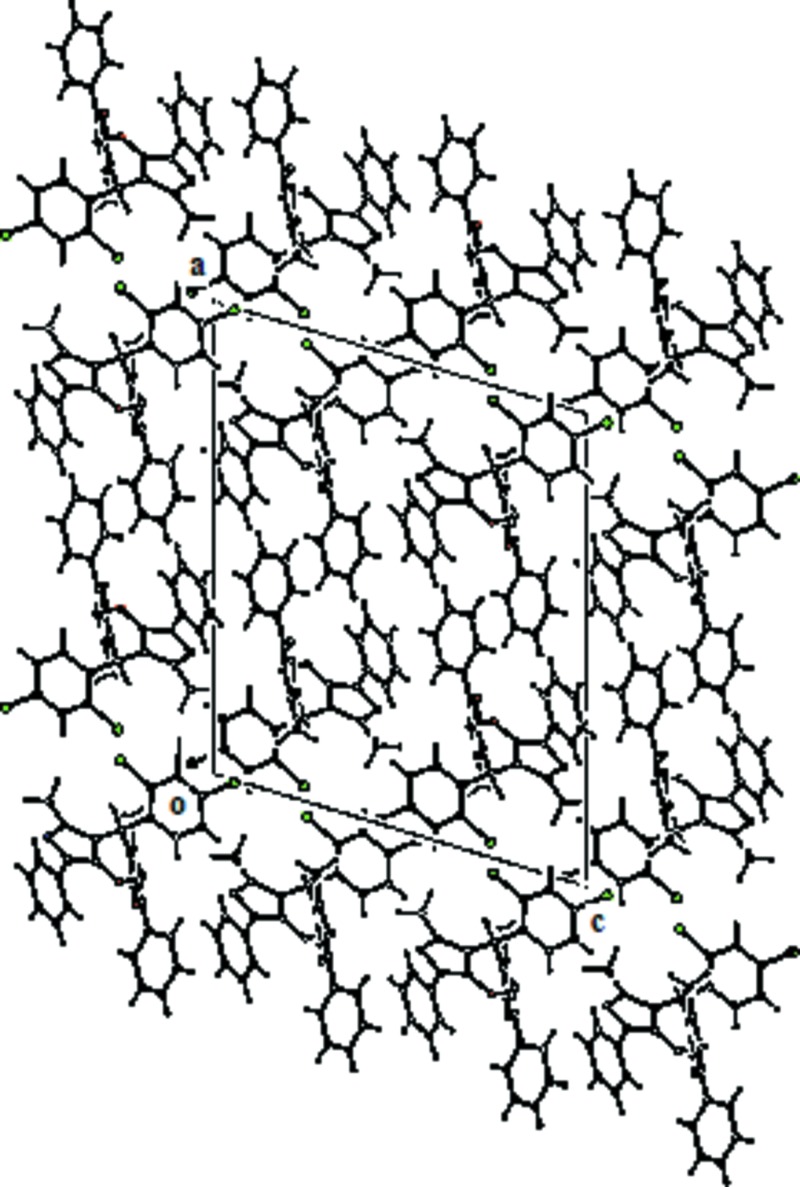
The packing arrangement of molecules viewed along the a axis.

ORTEP . DOI: 10.1107/S2056989015017880/sj5478fig1.tif
*ORTEP* view of the mol­ecule with the atom-labeling scheme. Displacement ellipsoids are drawn at the 40% probability level. H atoms are shown as small spheres of arbitrary radii.

a . DOI: 10.1107/S2056989015017880/sj5478fig2.tif
The packing arrangement of mol­ecules viewed along the *a* axis.

CCDC reference: 1427164

Additional supporting information:  crystallographic information; 3D view; checkCIF report

## Figures and Tables

**Table 1:** Hydrogen-bond geometry (, )

*D*H*A*	*D*H	H*A*	*D* *A*	*D*H*A*
N2H2O27^i^	0.86	2.08	2.756(3)	135
C24H24*A*Cl1^ii^	0.96	2.93	3.823(3)	156

Symmetry codes: (i) 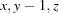; (ii) 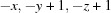.

## supplementary crystallographic information

### S1. Comment

Bis-pyrazolones are an important class of heterocyclic compounds which have risen to prominence in recent years due to their vital role in various biological activities such as selective COX-2 inhibition, antitumor and cytokine inhibition (Park *et al.*, 2005). Bis-pyrazolones are also used as antidepressants (Bailey *et al.*, 1985), gastric secretion stimulators (Rosiere & Grossman, 1951), or as antibacterial (Mahajan *et al.*, 1991) and antifilarial agents (Chauhan *et al.*, 1993). Moreover, 4,4'-(arylmethylene) bis(1*H*-pyrazol-5-ols) are used as pesticides, fungicides and dye stuffs (Hamama *et al.*, 2001). In recent years, different reagents have been reported for the synthesis of 4,4'-(arylmethylene)bis(3-methyl-1-phenyl-pyrazol-5-ol) derivatives including the condensation reaction between arylaldehyde and 5-methyl-2-phenyl-2,4-dihydro-3*H*-pyrazol-3-one (Niknam & Mirzaee, 2011; Bhardwaj *et al.*, 2015).

In the title compound C_27_H_22_C_12_N_4_O_2_, the pyrazole rings N1/N2/C3/C4/C5 and N6/N7/C8/C9/C10 make dihedral angle of 34.80 (11)° and 34.34 (12)° with the phenyl rings C18/C19/C20/C21/C22/C23 and C28/C29/C30/C31/C32/C33 respectively. The C5═O26 double bond [1.277 (3) Å] is significantly longer than that normally observed for carbonyl bonds [1.19 Å], probably because the hydroxyl group and carbonyl oxygen atom are involved in an intermolecular O—H···O hydrogen bond and is comparable with that found in a related structure (Sharma *et al.*, (2014). The bond lengths of C15—Cl1 [1.729 (4) Å] and C13—Cl2 [1.730 (4) Å], are comparable with the accepted value of 1.739 Å and are in good agreement with another molecule of this type (Sharma *et al.*, 2014). π - π interactions are observed between the pyrazole ring (N1/N2/C3/C4/C5) and phenyl ring (C28/C29/C30/C31/C32/C33) [centroid–centroid^i^ seperation = 3.916 (2) Å, interplanar spacing = 3.784 Å, centroid shift = 1.01 Å for symmetry operation: *x*, 1 + *y*, *z*]. Classical N–H···O and non-classical C–H···Cl hydrogen bonds, Table 1, also stabilise the crystal packing.

### S2. Experimental

Synthesis of 4-[(2,4-dichlorophenyl)(5-hydroxy-3-methyl-1-phenyl- 1*H*-pyrazol-4-yl)methyl]-1,2-dihydro-5-methyl-2-phenyl- 3*H*-pyrazol-3-one: To a mixture of 2,4-dichlorobenzaldehyde (1 mmol) and 5-methyl-2-phenyl-2, 4-dihydro-3*H*-pyrazol-3-one (2 mmol), melt of imidazole-DMU (30:70) was added and the reaction mixture was stirred at 70 ° C for the appropriate time. After completion of the reaction (monitored by TLC), the reaction mixture was cooled to room temperature and diluted with water (20 ml). The product was extracted with EtOAc (20 ml) and dried over anhydrous Na_2_SO_4_. Removal of the solvent under reduced pressure gave 4-[(2,4-dichlorophenyl)(5-hydroxy-3-methyl-1-phenyl-1*H*-pyrazol-4-yl) methyl]-1,2-dihydro-5-methyl-2-phenyl-3*H*-pyrazol-3-one which was purified by crystallization from ethyl acetate:pet ether. The product, was obtained as shiny white crystals.

### S3. Refinement

All the H atoms were fixed geometrically and allowed to ride on their parent C atoms, with C—H distances of 0.93–0.98 Å; and with *U*_iso_(H) = 1.2*U*_eq_(C), except for the methyl group where *U*_iso_(H) = 1.5*U*_eq_(C).

### Crystal data

**Table:**

C_27_H_22_Cl_2_N_4_O_2_	*F*(000) = 1048
*M**_r_* = 505.39	*D*_x_ = 1.377 Mg m^−^^3^
Monoclinic, *P*2_1_/*c*	Mo *K*α radiation, λ = 0.71073 Å
Hall symbol: -P 2ybc	Cell parameters from 2488 reflections
*a* = 19.8321 (19) Å	θ = 3.8–27.6°
*b* = 7.8574 (5) Å	µ = 0.30 mm^−^^1^
*c* = 16.3416 (16) Å	*T* = 293 K
β = 106.815 (10)°	Block, white
*V* = 2437.6 (4) Å^3^	0.30 × 0.20 × 0.20 mm
*Z* = 4	

### Data collection

**Table:**

Agilent Xcalibur, Sapphire3 diffractometer	4775 independent reflections
Radiation source: fine-focus sealed tube	2524 reflections with *I* > 2σ(*I*)
Graphite monochromator	*R*_int_ = 0.042
Detector resolution: 16.1049 pixels mm^-1^	θ_max_ = 26.0°, θ_min_ = 3.5°
ω scans	*h* = −24→11
Absorption correction: multi-scan (*CrysAlis PRO*; Agilent, 2013)	*k* = −9→8
*T*_min_ = 0.751, *T*_max_ = 1.000	*l* = −17→20
9479 measured reflections	

### Refinement

**Table:**

Refinement on *F*^2^	Primary atom site location: structure-invariant direct methods
Least-squares matrix: full	Secondary atom site location: difference Fourier map
*R*[*F*^2^ > 2σ(*F*^2^)] = 0.060	Hydrogen site location: inferred from neighbouring sites
*wR*(*F*^2^) = 0.174	H-atom parameters constrained
*S* = 1.00	*w* = 1/[σ^2^(*F*_o_^2^) + (0.0647*P*)^2^] where *P* = (*F*_o_^2^ + 2*F*_c_^2^)/3
4775 reflections	(Δ/σ)_max_ < 0.001
318 parameters	Δρ_max_ = 0.29 e Å^−^^3^
0 restraints	Δρ_min_ = −0.30 e Å^−^^3^

### Special details

**Table:**

Geometry. All e.s.d.'s (except the e.s.d. in the dihedral angle between two l.s. planes) are estimated using the full covariance matrix. The cell e.s.d.'s are taken into account individually in the estimation of e.s.d.'s in distances, angles and torsion angles; correlations between e.s.d.'s in cell parameters are only used when they are defined by crystal symmetry. An approximate (isotropic) treatment of cell e.s.d.'s is used for estimating e.s.d.'s involving l.s. planes.
Refinement. Refinement of *F*^2^ against ALL reflections. The weighted *R*-factor *wR* and goodness of fit *S* are based on *F*^2^, conventional *R*-factors *R* are based on *F*, with *F* set to zero for negative *F*^2^. The threshold expression of *F*^2^ > σ(*F*^2^) is used only for calculating *R*-factors(gt) *etc*. and is not relevant to the choice of reflections for refinement. *R*-factors based on *F*^2^ are statistically about twice as large as those based on *F*, and *R*- factors based on ALL data will be even larger.

### Fractional atomic coordinates and isotropic or equivalent isotropic displacement parameters (Å^2^)

**Table:**

	*x*	*y*	*z*	*U*_iso_*/*U*_eq_	
Cl1	0.00677 (6)	0.80461 (13)	0.44350 (7)	0.0924 (4)	
O27	0.28998 (11)	0.6772 (2)	0.75223 (13)	0.0473 (6)	
H27	0.2752	0.6135	0.7112	0.071*	
C10	0.26128 (16)	0.6342 (4)	0.81376 (18)	0.0403 (7)	
N2	0.26121 (14)	0.0048 (3)	0.69193 (16)	0.0483 (7)	
H2	0.2620	−0.1016	0.6800	0.058*	
O26	0.32799 (13)	0.4092 (3)	0.70772 (16)	0.0637 (7)	
N1	0.31308 (14)	0.1203 (3)	0.69305 (16)	0.0468 (7)	
C3	0.20859 (16)	0.0860 (4)	0.71276 (19)	0.0433 (7)	
C11	0.17730 (17)	0.3891 (4)	0.74490 (19)	0.0445 (7)	
H11	0.1429	0.3264	0.7659	0.053*	
N6	0.27580 (14)	0.7213 (3)	0.88777 (16)	0.0479 (7)	
C12	0.13422 (17)	0.4914 (4)	0.6682 (2)	0.0455 (8)	
C5	0.29086 (17)	0.2771 (4)	0.70900 (19)	0.0462 (8)	
C9	0.21443 (17)	0.5069 (3)	0.8164 (2)	0.0423 (7)	
C18	0.37530 (18)	0.0725 (4)	0.6727 (2)	0.0461 (8)	
N7	0.24029 (15)	0.6527 (4)	0.94179 (16)	0.0548 (7)	
C13	0.06910 (18)	0.5592 (4)	0.6645 (2)	0.0523 (8)	
C8	0.20479 (18)	0.5215 (4)	0.8983 (2)	0.0487 (8)	
C4	0.22364 (17)	0.2559 (4)	0.7224 (2)	0.0448 (8)	
C15	0.0563 (2)	0.6877 (4)	0.5296 (2)	0.0610 (10)	
C23	0.4379 (2)	0.1532 (4)	0.7111 (2)	0.0629 (10)	
H23	0.4394	0.2397	0.7504	0.075*	
C28	0.31742 (19)	0.8696 (4)	0.9144 (2)	0.0536 (9)	
C17	0.15898 (19)	0.5269 (4)	0.5982 (2)	0.0605 (9)	
H17	0.2025	0.4840	0.5974	0.073*	
C20	0.4358 (2)	−0.0977 (5)	0.5960 (2)	0.0745 (12)	
H20	0.4352	−0.1833	0.5564	0.089*	
C19	0.37351 (19)	−0.0548 (4)	0.6142 (2)	0.0592 (10)	
H19	0.3316	−0.1110	0.5875	0.071*	
C14	0.02955 (19)	0.6543 (4)	0.5963 (2)	0.0614 (10)	
H14	−0.0146	0.6951	0.5957	0.074*	
C24	0.14727 (19)	−0.0126 (4)	0.7233 (2)	0.0642 (10)	
H24A	0.1074	0.0038	0.6740	0.096*	
H24B	0.1359	0.0264	0.7734	0.096*	
H24C	0.1591	−0.1313	0.7293	0.096*	
C21	0.4975 (2)	−0.0175 (5)	0.6344 (3)	0.0718 (11)	
H21	0.5386	−0.0478	0.6214	0.086*	
C16	0.1201 (2)	0.6255 (5)	0.5291 (2)	0.0678 (10)	
H16	0.1379	0.6482	0.4834	0.081*	
C22	0.4984 (2)	0.1070 (5)	0.6918 (3)	0.0695 (10)	
H22	0.5405	0.1620	0.7186	0.083*	
C29	0.3772 (2)	0.8959 (5)	0.8911 (2)	0.0730 (11)	
H29	0.3916	0.8168	0.8573	0.088*	
C25	0.1636 (2)	0.4104 (5)	0.9406 (2)	0.0697 (11)	
H25A	0.1756	0.4378	1.0004	0.105*	
H25B	0.1748	0.2932	0.9340	0.105*	
H25C	0.1142	0.4288	0.9148	0.105*	
C30	0.4165 (2)	1.0431 (6)	0.9185 (3)	0.0920 (15)	
H30	0.4573	1.0621	0.9026	0.110*	
C33	0.2971 (3)	0.9859 (6)	0.9643 (3)	0.1041 (18)	
H33	0.2566	0.9676	0.9807	0.125*	
C31	0.3961 (3)	1.1586 (7)	0.9681 (3)	0.115 (2)	
H31	0.4226	1.2561	0.9867	0.138*	
C32	0.3362 (4)	1.1294 (7)	0.9901 (4)	0.143 (3)	
H32	0.3215	1.2088	1.0235	0.171*	
Cl2	0.03200 (6)	0.52304 (19)	0.74666 (7)	0.1010 (5)	

### Atomic displacement parameters (Å^2^)

**Table:**

	*U*^11^	*U*^22^	*U*^33^	*U*^12^	*U*^13^	*U*^23^
Cl1	0.0896 (9)	0.0657 (7)	0.0978 (8)	0.0008 (6)	−0.0113 (6)	0.0091 (6)
O27	0.0627 (15)	0.0263 (11)	0.0621 (13)	−0.0044 (10)	0.0328 (11)	−0.0077 (9)
C10	0.0476 (19)	0.0304 (16)	0.0489 (17)	0.0064 (14)	0.0233 (14)	0.0018 (14)
N2	0.0479 (17)	0.0221 (13)	0.0779 (18)	−0.0050 (11)	0.0232 (14)	−0.0084 (12)
O26	0.0655 (16)	0.0256 (11)	0.1200 (19)	−0.0075 (11)	0.0583 (14)	−0.0091 (12)
N1	0.0478 (17)	0.0259 (13)	0.0726 (17)	−0.0010 (12)	0.0269 (13)	−0.0013 (13)
C3	0.0413 (19)	0.0286 (16)	0.0617 (19)	0.0007 (14)	0.0177 (15)	−0.0025 (14)
C11	0.050 (2)	0.0267 (15)	0.065 (2)	−0.0010 (14)	0.0296 (15)	−0.0016 (14)
N6	0.0531 (18)	0.0389 (15)	0.0586 (17)	−0.0062 (13)	0.0270 (13)	−0.0058 (13)
C12	0.044 (2)	0.0288 (16)	0.067 (2)	−0.0038 (13)	0.0208 (15)	−0.0079 (15)
C5	0.050 (2)	0.0269 (15)	0.068 (2)	0.0014 (14)	0.0269 (16)	−0.0055 (15)
C9	0.0458 (19)	0.0253 (15)	0.0615 (19)	0.0004 (13)	0.0248 (14)	−0.0004 (14)
C18	0.052 (2)	0.0323 (16)	0.0582 (19)	0.0131 (14)	0.0227 (16)	−0.0026 (14)
N7	0.0557 (19)	0.0548 (18)	0.0604 (17)	−0.0054 (15)	0.0271 (14)	−0.0009 (14)
C13	0.042 (2)	0.0426 (18)	0.073 (2)	0.0023 (16)	0.0185 (17)	−0.0113 (17)
C8	0.049 (2)	0.0450 (19)	0.058 (2)	0.0062 (16)	0.0249 (16)	0.0054 (16)
C4	0.0420 (19)	0.0266 (15)	0.069 (2)	0.0015 (13)	0.0208 (15)	−0.0040 (14)
C15	0.055 (2)	0.0376 (19)	0.079 (3)	−0.0053 (17)	0.0003 (19)	−0.0054 (18)
C23	0.055 (2)	0.048 (2)	0.092 (3)	−0.0014 (18)	0.030 (2)	−0.0124 (19)
C28	0.054 (2)	0.048 (2)	0.0577 (19)	−0.0084 (17)	0.0153 (16)	−0.0079 (17)
C17	0.047 (2)	0.058 (2)	0.082 (2)	0.0052 (18)	0.0287 (19)	0.008 (2)
C20	0.079 (3)	0.071 (3)	0.079 (3)	0.027 (2)	0.031 (2)	−0.012 (2)
C19	0.054 (2)	0.056 (2)	0.065 (2)	0.0151 (18)	0.0126 (17)	−0.0104 (18)
C14	0.042 (2)	0.054 (2)	0.084 (3)	0.0018 (17)	0.0121 (19)	−0.017 (2)
C24	0.048 (2)	0.0358 (19)	0.111 (3)	−0.0119 (16)	0.026 (2)	−0.0087 (19)
C21	0.062 (3)	0.072 (3)	0.094 (3)	0.024 (2)	0.042 (2)	0.011 (2)
C16	0.066 (3)	0.063 (2)	0.076 (3)	0.003 (2)	0.025 (2)	0.013 (2)
C22	0.046 (2)	0.063 (2)	0.107 (3)	−0.0013 (19)	0.034 (2)	0.001 (2)
C29	0.064 (3)	0.078 (3)	0.086 (3)	−0.018 (2)	0.035 (2)	−0.009 (2)
C25	0.078 (3)	0.062 (2)	0.080 (2)	−0.006 (2)	0.041 (2)	0.014 (2)
C30	0.083 (3)	0.110 (4)	0.089 (3)	−0.057 (3)	0.033 (3)	−0.018 (3)
C33	0.110 (4)	0.097 (3)	0.131 (4)	−0.054 (3)	0.075 (3)	−0.067 (3)
C31	0.162 (6)	0.094 (4)	0.099 (4)	−0.077 (4)	0.053 (4)	−0.047 (3)
C32	0.179 (6)	0.119 (4)	0.166 (5)	−0.092 (5)	0.107 (5)	−0.093 (4)
Cl2	0.0630 (8)	0.1521 (12)	0.1022 (9)	0.0321 (7)	0.0465 (6)	0.0080 (8)

### Geometric parameters (Å, º)

**Table:**

Cl1—C15	1.729 (4)	C23—C22	1.375 (5)
O27—C10	1.334 (3)	C23—H23	0.9300
O27—H27	0.8200	C28—C33	1.361 (5)
C10—N6	1.346 (4)	C28—C29	1.362 (5)
C10—C9	1.375 (4)	C17—C16	1.401 (5)
N2—C3	1.348 (4)	C17—H17	0.9300
N2—N1	1.368 (3)	C20—C21	1.357 (5)
N2—H2	0.8600	C20—C19	1.395 (5)
O26—C5	1.277 (3)	C20—H20	0.9300
N1—C5	1.358 (4)	C19—H19	0.9300
N1—C18	1.418 (4)	C14—H14	0.9300
C3—C4	1.367 (4)	C24—H24A	0.9600
C3—C24	1.493 (4)	C24—H24B	0.9600
C11—C9	1.506 (4)	C24—H24C	0.9600
C11—C4	1.507 (4)	C21—C22	1.352 (5)
C11—C12	1.526 (4)	C21—H21	0.9300
C11—H11	0.9800	C16—H16	0.9300
N6—N7	1.388 (3)	C22—H22	0.9300
N6—C28	1.421 (4)	C29—C30	1.394 (5)
C12—C13	1.382 (4)	C29—H29	0.9300
C12—C17	1.397 (4)	C25—H25A	0.9600
C5—C4	1.422 (4)	C25—H25B	0.9600
C9—C8	1.410 (4)	C25—H25C	0.9600
C18—C23	1.374 (5)	C30—C31	1.354 (6)
C18—C19	1.377 (4)	C30—H30	0.9300
N7—C8	1.332 (4)	C33—C32	1.364 (6)
C13—C14	1.383 (5)	C33—H33	0.9300
C13—Cl2	1.730 (4)	C31—C32	1.356 (7)
C8—C25	1.495 (4)	C31—H31	0.9300
C15—C16	1.360 (5)	C32—H32	0.9300
C15—C14	1.368 (5)		
			
C10—O27—H27	109.5	C33—C28—N6	119.2 (3)
O27—C10—N6	121.4 (3)	C29—C28—N6	120.9 (3)
O27—C10—C9	130.4 (3)	C12—C17—C16	121.9 (3)
N6—C10—C9	108.2 (3)	C12—C17—H17	119.1
C3—N2—N1	108.5 (2)	C16—C17—H17	119.1
C3—N2—H2	125.8	C21—C20—C19	121.6 (4)
N1—N2—H2	125.8	C21—C20—H20	119.2
C5—N1—N2	108.6 (2)	C19—C20—H20	119.2
C5—N1—C18	129.6 (3)	C18—C19—C20	118.5 (4)
N2—N1—C18	121.5 (2)	C18—C19—H19	120.7
N2—C3—C4	109.4 (3)	C20—C19—H19	120.7
N2—C3—C24	120.0 (3)	C15—C14—C13	119.1 (4)
C4—C3—C24	130.6 (3)	C15—C14—H14	120.5
C9—C11—C4	114.9 (3)	C13—C14—H14	120.5
C9—C11—C12	110.2 (2)	C3—C24—H24A	109.5
C4—C11—C12	113.7 (2)	C3—C24—H24B	109.5
C9—C11—H11	105.7	H24A—C24—H24B	109.5
C4—C11—H11	105.7	C3—C24—H24C	109.5
C12—C11—H11	105.7	H24A—C24—H24C	109.5
C10—N6—N7	111.0 (2)	H24B—C24—H24C	109.5
C10—N6—C28	130.2 (3)	C22—C21—C20	119.1 (4)
N7—N6—C28	118.7 (3)	C22—C21—H21	120.4
C13—C12—C17	115.7 (3)	C20—C21—H21	120.4
C13—C12—C11	122.2 (3)	C15—C16—C17	119.3 (4)
C17—C12—C11	122.2 (3)	C15—C16—H16	120.3
O26—C5—N1	120.9 (3)	C17—C16—H16	120.3
O26—C5—C4	132.0 (3)	C21—C22—C23	120.9 (4)
N1—C5—C4	107.1 (3)	C21—C22—H22	119.6
C10—C9—C8	104.4 (3)	C23—C22—H22	119.6
C10—C9—C11	127.3 (3)	C28—C29—C30	119.2 (4)
C8—C9—C11	128.0 (3)	C28—C29—H29	120.4
C23—C18—C19	119.4 (3)	C30—C29—H29	120.4
C23—C18—N1	120.2 (3)	C8—C25—H25A	109.5
C19—C18—N1	120.4 (3)	C8—C25—H25B	109.5
C8—N7—N6	104.4 (2)	H25A—C25—H25B	109.5
C12—C13—C14	123.2 (3)	C8—C25—H25C	109.5
C12—C13—Cl2	120.3 (3)	H25A—C25—H25C	109.5
C14—C13—Cl2	116.4 (3)	H25B—C25—H25C	109.5
N7—C8—C9	111.9 (3)	C31—C30—C29	120.8 (4)
N7—C8—C25	118.7 (3)	C31—C30—H30	119.6
C9—C8—C25	129.4 (3)	C29—C30—H30	119.6
C3—C4—C5	106.2 (3)	C28—C33—C32	120.0 (5)
C3—C4—C11	125.3 (3)	C28—C33—H33	120.0
C5—C4—C11	128.4 (3)	C32—C33—H33	120.0
C16—C15—C14	120.8 (3)	C30—C31—C32	118.8 (4)
C16—C15—Cl1	119.8 (3)	C30—C31—H31	120.6
C14—C15—Cl1	119.4 (3)	C32—C31—H31	120.6
C18—C23—C22	120.4 (3)	C31—C32—C33	121.3 (5)
C18—C23—H23	119.8	C31—C32—H32	119.3
C22—C23—H23	119.8	C33—C32—H32	119.3
C33—C28—C29	119.9 (4)		
			
C3—N2—N1—C5	−4.1 (3)	C24—C3—C4—C5	176.8 (3)
C3—N2—N1—C18	−179.3 (3)	N2—C3—C4—C11	179.4 (3)
N1—N2—C3—C4	3.7 (3)	C24—C3—C4—C11	−1.9 (6)
N1—N2—C3—C24	−175.1 (3)	O26—C5—C4—C3	177.3 (3)
O27—C10—N6—N7	−179.6 (3)	N1—C5—C4—C3	−0.6 (3)
C9—C10—N6—N7	1.1 (3)	O26—C5—C4—C11	−4.1 (6)
O27—C10—N6—C28	3.5 (5)	N1—C5—C4—C11	178.0 (3)
C9—C10—N6—C28	−175.7 (3)	C9—C11—C4—C3	131.2 (3)
C9—C11—C12—C13	−79.3 (4)	C12—C11—C4—C3	−100.5 (4)
C4—C11—C12—C13	150.0 (3)	C9—C11—C4—C5	−47.1 (4)
C9—C11—C12—C17	98.7 (3)	C12—C11—C4—C5	81.1 (4)
C4—C11—C12—C17	−32.0 (4)	C19—C18—C23—C22	−0.4 (5)
N2—N1—C5—O26	−175.3 (3)	N1—C18—C23—C22	179.8 (3)
C18—N1—C5—O26	−0.6 (5)	C10—N6—C28—C33	144.3 (4)
N2—N1—C5—C4	2.9 (3)	N7—N6—C28—C33	−32.4 (5)
C18—N1—C5—C4	177.6 (3)	C10—N6—C28—C29	−36.1 (5)
O27—C10—C9—C8	178.6 (3)	N7—N6—C28—C29	147.3 (3)
N6—C10—C9—C8	−2.2 (3)	C13—C12—C17—C16	0.2 (5)
O27—C10—C9—C11	−6.1 (5)	C11—C12—C17—C16	−177.9 (3)
N6—C10—C9—C11	173.0 (3)	C23—C18—C19—C20	0.1 (5)
C4—C11—C9—C10	72.0 (4)	N1—C18—C19—C20	179.9 (3)
C12—C11—C9—C10	−57.9 (4)	C21—C20—C19—C18	0.1 (6)
C4—C11—C9—C8	−113.8 (4)	C16—C15—C14—C13	1.4 (5)
C12—C11—C9—C8	116.2 (3)	Cl1—C15—C14—C13	179.3 (2)
C5—N1—C18—C23	37.7 (5)	C12—C13—C14—C15	−1.7 (5)
N2—N1—C18—C23	−148.2 (3)	Cl2—C13—C14—C15	179.7 (3)
C5—N1—C18—C19	−142.1 (3)	C19—C20—C21—C22	0.0 (6)
N2—N1—C18—C19	32.0 (4)	C14—C15—C16—C17	−0.4 (6)
C10—N6—N7—C8	0.6 (3)	Cl1—C15—C16—C17	−178.2 (3)
C28—N6—N7—C8	177.8 (3)	C12—C17—C16—C15	−0.5 (6)
C17—C12—C13—C14	0.9 (5)	C20—C21—C22—C23	−0.3 (6)
C11—C12—C13—C14	179.0 (3)	C18—C23—C22—C21	0.5 (6)
C17—C12—C13—Cl2	179.4 (2)	C33—C28—C29—C30	−0.3 (6)
C11—C12—C13—Cl2	−2.5 (4)	N6—C28—C29—C30	180.0 (3)
N6—N7—C8—C9	−2.1 (4)	C28—C29—C30—C31	0.3 (7)
N6—N7—C8—C25	176.2 (3)	C29—C28—C33—C32	0.6 (7)
C10—C9—C8—N7	2.7 (4)	N6—C28—C33—C32	−179.7 (5)
C11—C9—C8—N7	−172.5 (3)	C29—C30—C31—C32	−0.5 (9)
C10—C9—C8—C25	−175.2 (3)	C30—C31—C32—C33	0.8 (10)
C11—C9—C8—C25	9.5 (6)	C28—C33—C32—C31	−0.9 (10)
N2—C3—C4—C5	−1.9 (4)		

### Hydrogen-bond geometry (Å, º)

**Table:**

*D*—H···*A*	*D*—H	H···*A*	*D*···*A*	*D*—H···*A*
N2—H2···O27^i^	0.86	2.08	2.756 (3)	135
C24—H24*A*···Cl1^ii^	0.96	2.93	3.823 (3)	156

Symmetry codes: (i) *x*, *y*−1, *z*; (ii) −*x*, −*y*+1, −*z*+1.
