# Supplementary figures and images for: Crystal structure of 4-[(2,4-di­chloro­phen­yl)(5-hy­droxy-3-methyl-1-phenyl-1H-pyrazol-4-yl)meth­yl]-5-methyl-2-phenyl-2,3-di­hydro-1H-pyrazol-3-one
Source: Acta Crystallogr E Crystallogr Commun. 2015 Sep 30;71(Pt 10):o805–6. doi: 10.1107/S2056989015017880 (PMC4647350; doi:10.1107/S2056989015017880)

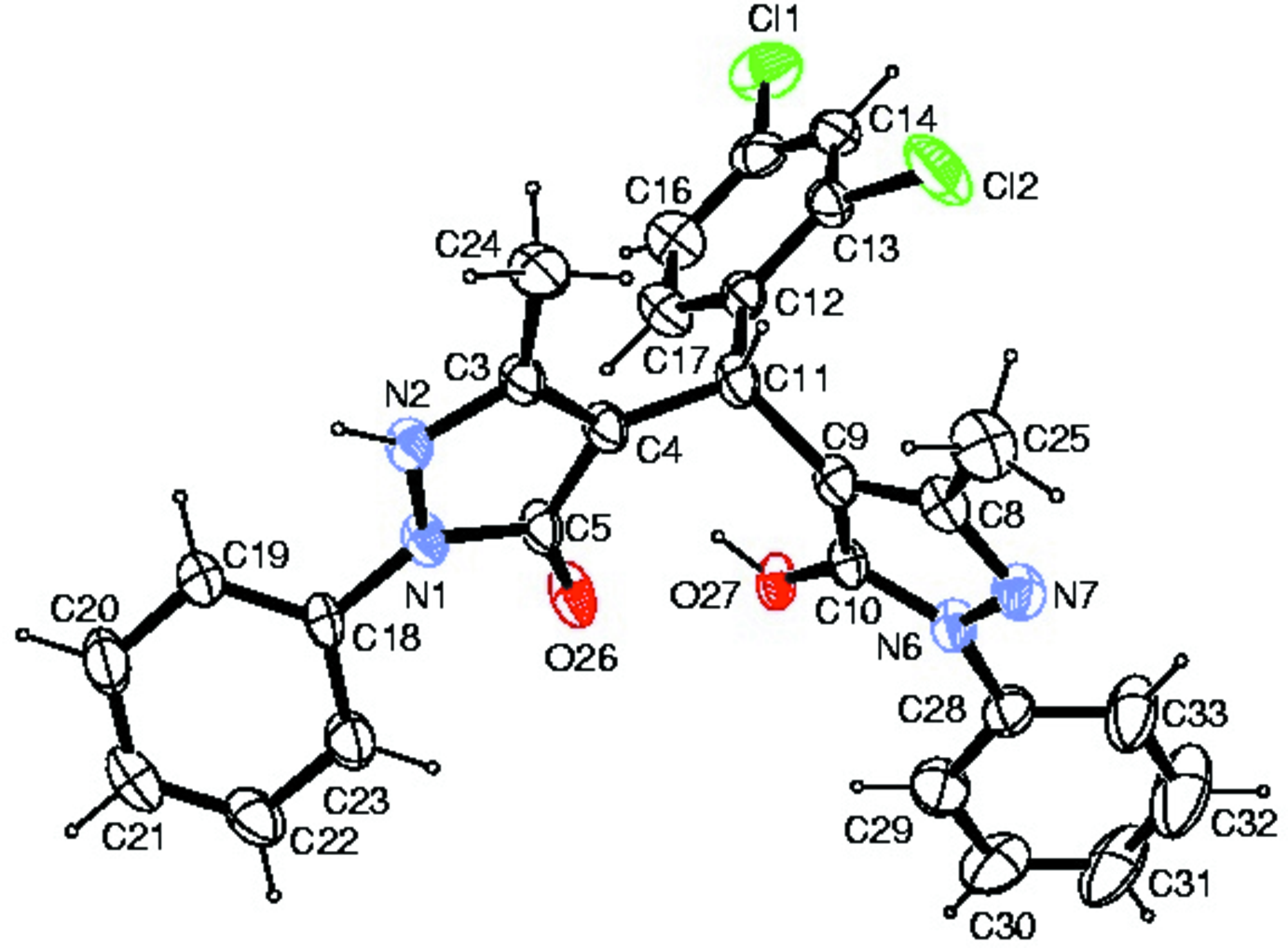

Supplement: Supplementary file 4 [file e-71-0o805-fig1.tif]

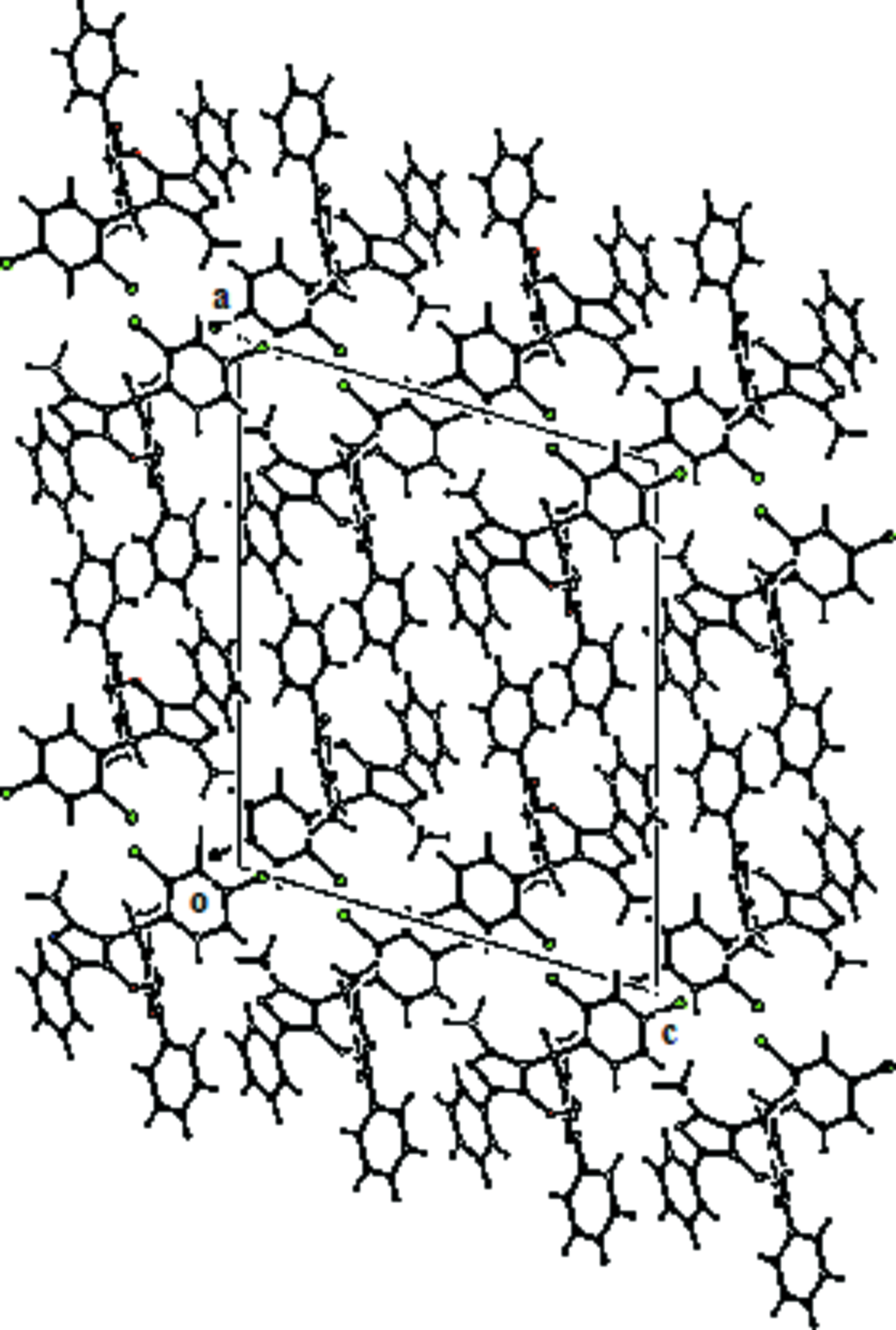

Supplement: Supplementary file 5 [file e-71-0o805-fig2.tif]
